# Cardiovascular risk moderates the effect of aerobic exercise on executive functions in older adults with subcortical ischemic vascular cognitive impairment

**DOI:** 10.1038/s41598-021-99249-1

**Published:** 2021-10-07

**Authors:** Cindy K. Barha, Elizabeth Dao, Lauren Marcotte, Ging-Yuek Robin Hsiung, Roger Tam, Teresa Liu-Ambrose

**Affiliations:** 1 Aging, Mobility, and Cognitive Neuroscience Lab, Department of Physical Therapy, University of British Columbia, Vancouver, Canada; 2 Djavad Mowafaghian Centre for Brain Health, 2215 Wesbrook Mall, Vancouver, BC V6T 2B5 Canada; 3 Division of Neurology, University of British Columbia, Vancouver, Canada; 4 Vancouver Coastal Health Research Institute and University of British Columbia Hospital Clinic for Alzheimer Disease and Related Disorders, Vancouver, Canada; 5 Department of Radiology, University of British Columbia, Vancouver, Canada; 6 Centre for Hip Health and Mobility, Vancouver, Canada; 7 School of Biomedical Engineering, University of British Columbia, Vancouver, Canada

**Keywords:** Dementia, Clinical trial design

## Abstract

Aerobic training (AT) can promote cognitive function in adults with Subcortical Ischemic Vascular Cognitive Impairment (SIVCI) by modifying cardiovascular risk factors. However, pre-existing cardiovascular health may attenuate the benefits of AT on cognitive outcomes in SIVCI. We examined whether baseline cardiovascular risk moderates the effect of a 6-month progressive AT program on executive functions with a secondary analysis of a randomized controlled trial in 71 adults, who were randomized to either: (1) 3×/week progressive AT; or (2) education program (CON). Three executive processes were measured: (1) response inhibition by Stroop Test; (2) working memory by digits backward test; and (3) set shifting by the Trail Making Test. Baseline cardiovascular risk was calculated using the Framingham cardiovascular disease (CVD) Risk Score (FCRS), and participants were classified as either low risk (< 20% FCRS score; LCVR) or high risk (≥ 20% FCRS score; HCVR). A complete case analysis (n = 58) was conducted using an analysis of covariance (ANCOVA) to evaluate between-group differences in the three executive processes. A significant interaction was found between cardiovascular risk group and intervention group (AT or CON) for the digit span backward and the Trail Making Test. AT improved performance compared with CON in those with LCVR, while in those with HCVR, AT did not improve performance compared with CON. Baseline cardiovascular risk significantly moderates the efficacy of AT on cognition. Our findings highlight the importance of intervening early in the disease course of SIVCI, when cardiovascular risk may be lower, to reap maximum benefits of aerobic exercise.

## Introduction

Cardiovascular risk factors, including hypertension, dyslipidemia, diabetes, and smoking, are associated with cognitive decline over time and increased dementia risk^1–3^. Thus, these modifiable risk factors are targets for promoting healthy cognitive aging and prevention of dementia. Engaging in physical exercise is a promising primary behavioural activity that reduces key cardiometabolic risk factors^4^ and protects cognition in older age^5–7^.

Vascular cognitive impairment is the second most common form of dementia after Alzheimer’s disease and is associated with or caused by cerebrovascular disease^8^. Subcortical ischemic vascular cognitive impairment (SIVCI) is the most common form of vascular cognitive impairment and is clinically defined by cognitive impairment and the presence of subcortical vascular brain injury, including lacunar infarcts, white matter hyperintensities, and microbleeds^9,10^. Additionally, arteriolosclerosis, dilation of perivascular spaces, venous collagenosis, and cerebral amyloid angiopathy can be found in the brains of SIVCI patients^11^. These pathophysiological changes to the white matter which are most severe in the frontal and occipital regions^12,13^, are predictive of cognitive impairment^14^. While patients with SIVCI show deficits across all cognitive domains, deficits are most pronounced in executive functions^15^ which are subserved by the prefrontal cortex. Cardiovascular risk factors are intimately associated with SIVCI^16^ and thus, reductions in and control of cardiovascular risk factors are vital for the successful management of SIVCI.

Exercise is a potentially effective primary and secondary intervention for SIVCI^17^. Moderate to high intensity levels of physical activity were associated with lower volume of white matter hyperintensities in older healthy adults^18^. Furthermore, engaging in resistance training also reduced white matter hyperintensity progression in older adults^19,20^. In the only randomized controlled trial (RCT) conducted in older adults with SIVCI, 6 months of aerobic training (AT) led to improvements in global cognitive function and reductions in diastolic blood pressure^17^. Additionally, a secondary analysis of this study indicated AT improved executive functions in the female participants^21^; impaired executive functions are common in SIVCI^22^.

Mounting evidence suggests AT may promote cognition in SIVCI by modifying cardiovascular risk factors, including diabetes, cholesterol, and hypertension^23–25^. Previous work has shown that AT improved the cardiovascular risk factor of high diastolic blood pressure in those with SIVCI^17^ and others have shown that AT can directly influence these cardiometabolic risk factors^26–30^. However, it is currently not known whether one’s cardiovascular health may attenuate the benefits of AT on cognitive outcomes in SIVCI. In the context of SIVCI, cardiovascular disease (CVD) risk predicted vascular cognitive impairment diagnosis and lower global cognition scores^31^. These findings suggest that AT-induced cognitive benefits in older adults with SIVCI may be moderated by cardiovascular risk profiles.

Therefore, we conducted a secondary analysis of data collected from a proof-of-concept, single-blind RCT (NCT01027858—PROMoTE Study) of three times per week progressive AT in older adults with SIVCI^17,32^ to examine whether baseline cardiovascular risk can moderate the effect of AT on executive functions. We hypothesized that the beneficial effect of AT compared with control would be dependent on cardiovascular risk profiles.

## Methods

### Study design

This is a secondary analysis of a 26-week, single-blind RCT of progressive AT in 71 older adults with clinically confirmed SIVCI. The design and primary results of the RCT have been previously reported^17,32^. Briefly, physical and cognitive assessments for all participants were conducted at baseline and at trial completion at 6-months post-randomization. Eligible participants meeting study inclusion and exclusion criteria were randomized into either the 6-month progressive AT group or the usual care plus education control (CON) group. Ethics approval was obtained from the Clinical Research Ethics Board at the University of British Columbia (H07-01160) and Vancouver Coastal Health Research Institute (V07-01160), and trial protocol was registered at ClinicalTrials.gov (NCT01027858). All research was performed in accordance with the relevant guidelines and regulations of the institutions involved and in accordance with the Declaration of Helsinki. Informed consent was obtained from all participants.

### Participants

Participants were recruited from the University of British Columbia Hospital Clinic for Alzheimer’s Disease and Related Disorders, the Vancouver General Hospital Stroke Prevention Clinic, and specialized geriatric clinics in Vancouver, British Columbia. A detailed description of the inclusion and exclusion criteria has been previously published^17,32^. Briefly, individuals were eligible for study entry if they met the following criteria: (1) diagnosed with SIVCI^33^ as confirmed by a neurologist based on the presence of cerebral small vessel disease, defined as the presence of periventricular or deep white matter lesions and the absence of non-lacunar territorial (cortical and/or cortico-subcortical) strokes on clinical computerized tomography or magnetic resonance imaging (MRI) scans, and mild cognitive impairment, defined as a Montreal Cognitive Assessment (MoCA) score of < 26/30^34^; (2) aged 55 years or older; (3) Mini-Mental State Examination (MMSE) score of ≥ 20 at screening^35^; and (4) provide informed consent. Individuals were excluded from participating in the RCT if they were: (1) diagnosed with dementia of any type or other neurological conditions; (2) taking medications that may negatively affect cognitive function (e.g., anticholinergics); and (3) planning on concurrently participating in a clinical drug trial.

### Measurements

#### Descriptive variables

At baseline, age, biological sex, education level, weight (kg), and waist-to-hip ratio (WHR = waist circumference/hip circumference) were collected. Global cognition was measured with the MMSE and MoCA. The Functional Comorbidity Index (FCI) assessed the number of comorbid conditions related to physical functioning^36^, the Short Physical Performance Battery (SPPB) assessed general mobility and balance^37^, the Timed Up-and-Go (TUG) Test assessed functional mobility^38^, the 15-item Geriatric Depression Scale (GDS) screened for depression^39^, and the Physical Activity Scale for the Elderly (PASE) assessed self-reported physical activity levels^40^.

#### Executive functions

Three executive functions were assessed in this secondary analysis: working memory, set-shifting, and response inhibition. These three specific executive processes were included based on the work of Miyake et al.^41^ and the high frequency of inclusion in clinical batteries^42^.

Working memory was assessed with the verbal digits backward test (Digits-B)^43^. Briefly, seven pairs of random number sequences were read aloud by the assessor at a rate of one per second, starting with sequences that are three digits in length and increasing by one digit each time to a maximum sequence length of nine digits. Two pairs of sequences of each length were given and the test was terminated once the participant failed to recollect any two sequences of the same length. The participant was required to repeat the sequence in reverse order. The final score was the number of successful sequences recalled in reverse order (range 0–14) by the participant, with higher scores indicating better performance.

Set-shifting was assessed with the Trail-Making tests (Part B minus Part A; TMT_B–A_)^44^. Participants were required to draw lines between encircled numbers connecting them in ascending order (Part A) and connecting alternating numbers and letters in ascending order (Part B). Errors were recorded for each participant and when an error was made, the participant’s attention was drawn to the error and they were instructed to proceed from the point the error was made. The timer was not stopped. The amount of time in seconds needed to complete each part was recorded for each participant and set-shifting was indexed by calculating the difference in completion time between Part B and Part A, with smaller scores indicating better set-shifting ability.

Response inhibition was assessed with the Stroop Test^45^. Participants were required to first read aloud words printed in black ink. Then in the congruent trials, participants said aloud the ink color that X’s were printed in. Then in the incongruent trials, participants were asked to say aloud the ink color in which words were printed in while simultaneously ignoring the written word itself. The time taken to complete each trial was recorded and response inhibition was calculated as the time difference between the incongruent and congruent trials, with smaller scores indicating better performance.

#### Cardiovascular risk

Several models have been developed to assess an individual’s risk of CVD, with the Framingham General CVD Risk Score (FCRS) being the most extensively used in both clinical and research settings^46^. The FCRS has been shown to have good accuracy in predicting risk for dementia and cognitive decline, with poorer FCRS associated with adverse cognitive outcomes^47^. Baseline data were used to calculate each participant’s risk of developing any CVD event (coronary death, myocardial infarction, coronary insufficiency, angina, ischemic stroke, hemorrhagic stroke, transient ischemic attack, peripheral artery disease, heart failure) based on the Framingham Heart Study Cardiovascular Disease 10-year Risk Profile^46^. Specifically, the predictors included in the multivariable risk algorithm to calculate each participant’s FCRS were: sex, age (years), systolic blood pressure (mmHg), treatment for hypertension (yes, no), current smoker (yes, no), diabetes (yes, no), total cholesterol, and high-density lipoprotein cholesterol. The resulting FCRS for each participant estimates the 10-year probability of developing any CVD that is expressed as a percent.

#### General cardiovascular capacity

The 6-Minute Walk Test (6MWT) assessed general cardiovascular capacity^48^. For each participant, the meters walked in 6 min was recorded at baseline and trial completion.

### Experimental groups

#### Aerobic training group (AT)

All AT group classes were led by certified exercise instructors, were 60 min in duration (10-min warmup, 40-min walking program, 10-min cool down), and occurred 3 times per week for 26 weeks. Class attendance was monitored and recorded by the instructors. Compliance was calculated as the percentage of the total classes attended by each participant. The walking program consisted of following a predetermined outdoor route around the local neighborhood and was progressive in intensity. Participant’s intensity was monitored and progressed using two techniques: (1) a heart rate monitor was worn and initially participants walked at approximately 40% of their sex and age specific heart rate reserve (HRR). AT intensity was slowly progressed over the first 12 weeks to between 60 and 70% HRR, with a target of 65% HRR that was maintained for the remainder of the trial; (2) the 20-point Borg Rating of Perceived Exertion (RPE)^49^ was used to subjectively monitor the AT intensity, which was initially set at 11 and progressed to a target of 14–15 (“hard”); (3) the simple “talk” test was also used to subjectively monitor and progress AT intensity, with participants initially asked to walk at a pace that allowed them to converse comfortably and gradually progressed to a pace where conversation required effort.

#### Usual care plus education control group (CON)

Control participants attended monthly education classes and were given material about vascular cognitive impairment and healthy diet. Information regarding exercise and physical activity were not provided. Research staff also phoned participants each month to maintain contact and collect data.

### Statistical analyses

All statistical analyses were performed using the Statistical Package for the Social Sciences 24.0 (IBM Corporation Armonk, NY, USA). Baseline cardiovascular risk was calculated using the FCRS, and participants were classified as either low cardiovascular risk (< 20% FCRS; LCVR) or high cardiovascular risk (≥ 20% FCRS; HCVR). Analyses were conducted on complete cases (n = 58), including only those participants with FCRS.

An analysis of covariance (ANCOVA) was conducted to evaluate the main effects and interaction between experimental group (AT, CON) and cardiovascular risk group (LCVR, HCVR), controlling for age, baseline MoCA score, education, and baseline score for the outcome variable of interest. Separate ANCOVAs were run for the three executive functions outcomes—Digits-B, TMT_B–A_, and Stroop. The overall alpha was set at 0.05 and effect sizes for significant results were calculated as partial eta squared (η_p_^2^).

## Results

### Participants and sample descriptive

Complete case analyses were conducted on 58 of the 71 randomized participants. After randomization, one participant was deemed ineligible due to the diagnosis of mixed dementia and was excluded from all analyses. Of the 58 complete cases, 32 were in the AT group and 26 were in the CON group. The subsample of complete cases did not significantly differ from the parent sample of 70 on baseline characteristics such as age, WHR, MMSE, MoCA, comorbidities, SPPB, TUG, 6MWT and GDS.

Table 1 reports the baseline descriptive characteristics of the 58 complete case participants separated by experimental group and cardiovascular risk group.

**Table 1:** Baseline descriptive characteristics across intervention and cardiovascular risk groups.

Variables	Aerobic Training group, mean (SD)		Control group, mean (SD)	
	LCVR, n = 21	HCVR, n = 11	LCVR, n = 12	HCVR, n = 14
Age	72.95 (8.32)	75.82 (6.82)	70.25 (7.44)	76.36 (6.31)
Education (> high school)	21	10	11	14
Female	16	2	7	5
Montreal Cognitive Assessment	21.05 (2.99)	21.00 (3.19)	23.08 (2.28)	22.64 (3.41)
Mini-Mental State Examination	27.00 (2.57)	25.18 (2.60)	27.58 (1.56)	26.71 (2.95)
Timed up and go	8.50 (2.40)	8.14 (1.12)	7.63 (1.22)	8.92 (3.00)
Short physical performance battery	10.68 (1.60)	11.36 (1.21)	11.08 (0.67)	10.14 (1.29)
Waist to hip ratio	0.85 (0.07)	0.92 (0.08)	0.89 (0.07)	0.95 (0.06)
Functional Comorbidity Index	3.19 (1.63)	3.36 (1.36)	3.08 (1.98)	4.00 (2.00)
Six Minute Walk Test (m)	524.25 (101.81)	507.73 (72.22)	534.83 (80.39)	463.21 (101.43)
Geriatric Depression Scale	2.24 (2.59)	1.82 (1.89)	1.75 (1.42)	2.57 (2.38)
Physical Activity Scale for the Elderly	124.98 (69.24)	130.82 (91.55)	141.58 (44.69)	121.90 (61.22)

### Compliance

The average compliance in the AT group for this complete case sample was 69.4%. Compliance did not differ between the LCVR and HCVR AT participants [F(1,30) = 0.002, p > 0.690; 69.5% vs. 69.2%, respectively].

### Executive functions

**Figure 1:**
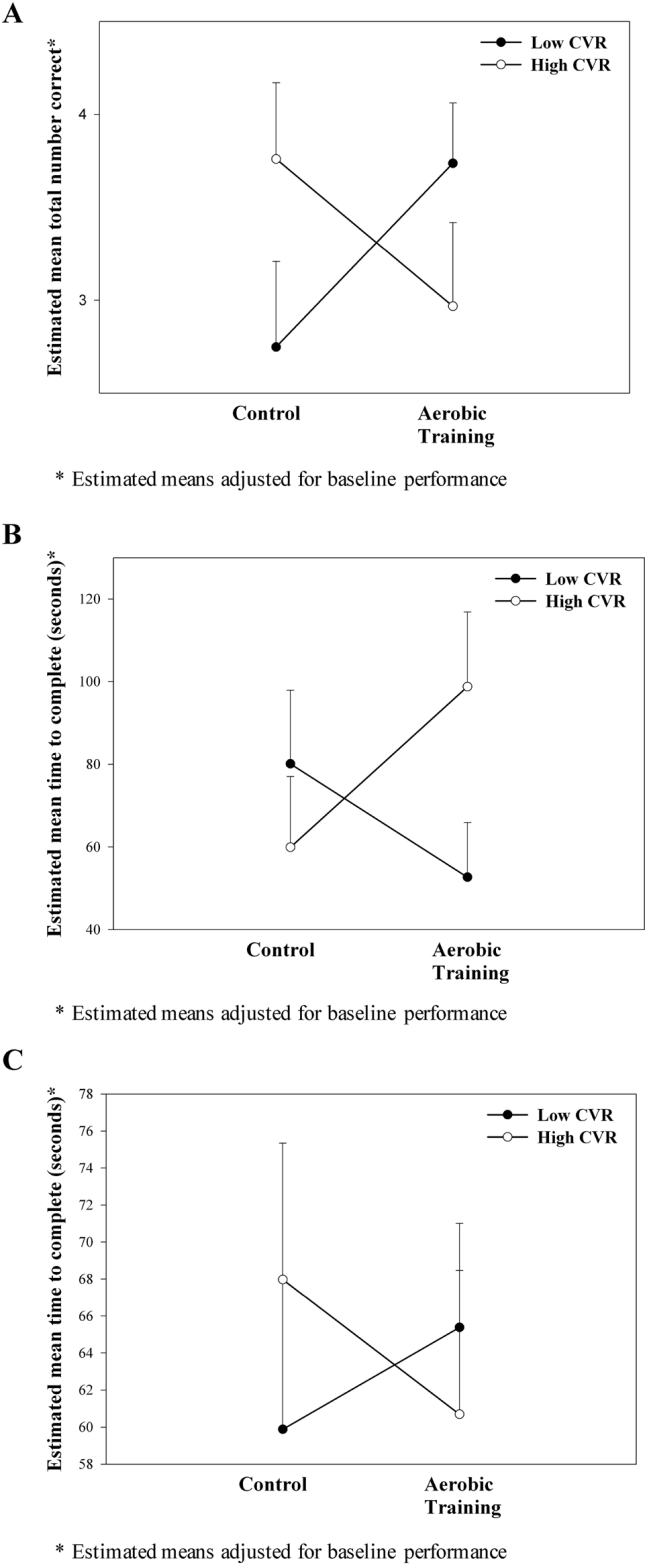
Interaction between cardiovascular risk (CVR) group (low CVR, high CVR) and experimental group (control, aerobic training) on the three tests of executive function at trial completion, controlling for baseline performance on the test, age, baseline MoCA score, and education. (**A**) Estimated mean (+ SEM) total number correct on the Verbal digits backward test at trial completion, with a significant interaction between cardiovascular risk group and experimental group (p < 0.037), suggesting that aerobic training improved the total number of correct responses compared to control in the low CVR participants but not in the high CVR participants. (**B**) Estimated mean (+ SEM) time to complete the Trail-Making tests (Part B minus Part A; seconds) at trial completion, with a significant interaction between cardiovascular risk group and experimental group (p < 0.048), suggesting that aerobic training improved performance compared to control in the low CVR participants but not in the high CVR participants. (**C**) Estimated mean (+ SEM) time to complete the Stroop test at trial completion, with no significant interaction between cardiovascular risk group and experimental group (p > 0.819), suggesting that aerobic training not influence performance compared to control in either the low CVR or high CVR participants.

Figure 1 presents the scores on the 3 tests of executive functions at trial completion for LCVR and HCVR participants in the CON and AT groups.

For the Digits-B test, a significant interaction was found between experimental group and CVR group [F(1,49) = 4.670, p < 0.037, η_p_^2^ = 0.087], after controlling for age, baseline MoCA score, education, and baseline Digits-B score, with AT improving performance compared to CON in the LCVR participants but not in the HCVR participants (see Fig. 1A). Main effects of experimental group [F(1,49) = 0.055, p > 0.816] or CVR group [F(1,49) = 0.081, p > 0.777] were not found.

For TMT_B–A_, a significant interaction was found between experimental group and CVR group [F(1,50) = 4.091, p < 0.048, η_p_^2^ = 0.076], after controlling for age, baseline MoCA score, education, and baseline TMT_B-A_ score, with AT improving performance compared to CON in the LCVR participants but not in the HCVR participants (see Fig. 1B). Main effects of experimental group [F(1,50) = 0.111, p > 0.740] or CVR group [F(1,50) = 0.570, p > 0.454] were not found.

For Stroop, main effects of experimental group [F(1,48) = 0.015, p > 0.905] or CVR group [F(1,48) = 0.053, p > 0.819] were not found. Neither was an interaction between experimental group and CVR group [F(1,48) = 0.802, p > 0.375], after controlling for age, baseline MoCA score, education, and baseline Stroop score (see Fig. 1C).

### Cardiovascular capacity and fitness

The 6MWT, which assessed general cardiovascular capacity, at trial completion did not differ between the four groups [F(1,46) = 1.236, p > 0.271] (AT LCVR: 535.93 ± 12.16 m; CON LCVR: 522.80 ± 15.47 m; AT HCVR: 550.45 ± 16.56 m; CON HCVR: 505.22 ± 15.14 m).

## Discussion

The present study found that baseline cardiovascular risk, assessed by the FCRS, moderated the efficacy of aerobic exercise on working memory and set shifting in adults with SIVCI. Specifically, the beneficial effects of 6-months of moderate-intensity AT were dependent on baseline cardiovascular risk, with our results suggesting AT was associated with better executive functions in those with a low cardiovascular risk. To our knowledge, no prior exercise trial in adults with SIVCI has shown this. These findings highlight the importance of intervening early in the disease course of SIVCI, when cardiovascular risk may be lower, to reap maximum benefits of aerobic exercise.

Previously, 6-months of moderate-intensity AT was shown to improve general cognition in adults with SIVCI^17^. We extend these findings, showing that cardiovascular health is an important moderator of AT efficacy in SIVCI. Somewhat surprisingly, we found that AT was more effective in improving executive functions in those with low cardiovascular risk than those with high risk, even though studies in other populations suggest that exercise can reduce key cardiovascular risk factors associated with SIVCI^50^. Our current RCT findings align with a population-based cohort study that showed physical activity was more efficacious in preventing mild cognitive impairment among those without cardiovascular risk factors such as high cholesterol, smoking history, and alcohol use^51^. Interestingly, Fig. 1 suggests that AT may actually impair performance on 2 of the 3 executive functions tasks in the high cardiovascular risk group, indicating that pre-existing unhealthy cardiovascular and cerebrovascular systems may not respond positively to moderate intensity AT. Future trials powered to detect subgroup differences based on cardiovascular risk are warranted.

The lack of significant effect of AT on executive functions in participants with high cardiovascular risk, while surprising, could be due to several factors. The current study utilized a 6-month, three times per week moderate intensity AT protocol. It may be the case that in adults with SIVCI and high cardiovascular risk, exercise protocols utilizing different parameters, such as higher intensity or longer duration, are needed. For example, low volume, high intensity AT has been shown to improve several indices of cardiometabolic health in obese participants^52^. Further, a recent meta-analysis comparing high intensity interval training vs. moderate intensity continuous training found that high intensity interval training was more effective in improving cardiorespiratory fitness and cardiovascular health in subgroups of participants at high risk, including those with type 2 diabetes and metabolic syndrome, and obese participants^53^. Furthermore, the 2018 consensus statement from the EXercise Prescription in Everyday Practice & Rehabilitative Training (EXPERT) working group endorsed by the European Association of Preventative Cardiology concluded that specific recommendations on exercise prescription including exercise type, volume, frequency, and intensity, should be based on different combinations of cardiovascular disease risk factors and may be different for those at low vs. high risk of CVD^54^. Importantly, the current analysis did not find that the AT intervention improved cardiovascular fitness differently in those at low versus high cardiovascular fitness, suggesting that the greater AT impact on executive functions in low risk individuals was independent of cardiovascular fitness. Greater changes in fitness level may be required in those at high cardiovascular risk in order to observe changes in cognition. In addition to AT, resistance training is a viable alternative in those at higher cardiovascular risk^55^, particularly as some evidence suggests that higher Framingham risk scores are associated with greater development of impaired mobility in older adults^56^ that could impair their ability to reap maximal benefits from AT paradigms. The findings of the current study in combination with previous findings, highlight that “one size does not fit all” and there is a need for further research to identify important moderators of exercise efficacy and provide more precise recommendations for specific groups of individuals.

Our results support the importance of intervening earlier in the disease course of SIVCI and before cardiovascular risk is high. Importantly, midlife has been proposed to be a critical window for implementing exercise interventions to improve cognitive health^57^. Physical fitness during midlife is a strong predictor of cardiovascular health in older age^58^ and an increasing body of evidence indicates that engaging in exercise during midlife can improve cardiovascular outcomes^59^. Thus future trials should focus on exercise interventions conducted during the middle years of life in order to promote cognitive aging through improved cardiovascular health. As well, future research is required to further examine how AT is influencing specific executive processes but not others. Specifically, we found that AT was influencing working memory and set-shifting but not response inhibition. According to Miyake et al.^41^, while these 3 executive functions are related and unified, they are also diverse and distinct from each other. Further research is required in this area to determine how these executive functions are similar and distinct and how they are further influenced by exercise.

The findings of the current analyses should be evaluated within the context of the study limitations. This secondary analysis of data from complete cases is limited by the small sub-group sample size. Thus, these findings are preliminary and we did not run posthoc comparisons between groups to avoid multiple comparisons. Cardiovascular risk was evaluated using the widely accepted Framingham Heart Study Cardiovascular Disease 10-year Risk Profile^46^ and high cardiovascular risk was determined using the established clinical cut-off of 20% or higher^60^. While the Framingham risk profile is an accepted and valid model, other risk models have been proposed including the CAIDE model^61^, the vascular index^62^, the vascularity index^63^, the atherosclerotic risk profile^64^, the hypoperfusion risk profile^64^, and the cardiovascular composite model^1^. The utility of these models for predicting cognitive decline and dementia has been established^47^. And while these models are superior to using single cardiovascular risk factors such as cholesterol levels, determining which specific risk model is best suited to specific populations within the context of exercise intervention efficacy has yet to be examined. For example Klages et al.^63^ found that the vascularity index was not associated with risk of Alzheimer’s disease over 5 years but was associated with risk of vascular cognitive impairment, thus this type of model may be more suitable to determine cardiovascular risk in a SIVCI sample, though the participants in the current study were diagnosed with a mild form of SIVCI. It is also plausible that high cardiovascular risk may be indicative of more advanced SIVCI then low risk. We did not control for brain pathologies, thus AT may have been more efficacious in the low CVR group which may have had less brain pathology. Thus, it would be of great interest in studies with larger sizes to examine the potential moderation of the relationship between cardiovascular risk, exercise, and cognition by SIVCI stage. Furthermore, very few females in our sample were categorized as being high cardiovascular risk. This may be due to the use of the Framingham risk profile and the specific biological variables used by this measure or more likely related to the need to establish different cutoffs for risk for males versus females^65^. Further research in this area looking at potential sex differences is highly warranted.

## Conclusion

In summary, this secondary analysis of data from a proof-of-concept RCT found that baseline cardiovascular risk significantly moderates the efficacy of aerobic exercise on working memory and set shifting in adults with SIVCI. Future trials should include a more comprehensive examination of these two executive function processes as the present study utilized single tests to assess each process. These findings highlight the importance of intervening early in the disease course of SIVCI, when cardiovascular risk may be lower, to reap maximum benefits of aerobic exercise. Additionally, the results suggest that cardiovascular health is a potentially important variable that may help identify target populations for which ‘exercise is medicine’ for promoting and maintaining brain health.
